# Metacognition in Argumentative Writing Based on Multiple Sources in Geography Education

**DOI:** 10.3390/ejihpe12080069

**Published:** 2022-08-01

**Authors:** Diana Gebele, Alexandra L. Zepter, Pia Königs, Alexandra Budke

**Affiliations:** 1 Department of German Language and Literature II, Faculty of Arts and Humanities, University of Cologne, 50923 Cologne, Germany; azepter@uni-koeln.de (A.L.Z.); pia.koenigs@uni-koeln.de (P.K.); 2 Institute of Geography Education, Faculty of Mathematics and Natural Sciences, University of Cologne, 50923 Cologne, Germany; alexandra.budke@uni-koeln.de

**Keywords:** metacognition, argumentative writing based on multiple sources, material-based argumentative writing, reading, geography teaching

## Abstract

This paper addresses questions about the use of metacognitive strategies in argumentative writing based on multiple sources and the influence of this use on the quality of student texts. For this purpose, think-aloud protocols and texts from a research project on material-supported argumentative writing in 8th grade geography lessons are analyzed and discussed. The analysis is based on a model of metacognition in argumentative writing using multiple sources, which we also propose in this paper. The results show that the use of metacognitive strategies is a challenge for the investigated target group but that their use, in particular the deployment of goal-setting strategies and planning strategies, enables students to write better texts.

## 1. Introduction

Subject didactics and psychology research on cognitive learning are necessarily focused on the various factors influencing the success of learning processes. In this context, research has recently given more attention to metacognition as an important variable that influences learning. The term metacognitive refers to being aware of one’s own knowledge as well as thinking strategies and consciously regulating them [1] (p. 130). Central to the basic understanding of metacognitive aspects or categories is that, by definition, they concern a meta-level of cognition, either in that they involve cognitive knowledge, experiences of one’s own cognitive activities, or cognitive control processes.

The development of metacognitive theory in the late 1970s [2,3,4] played a role in far-reaching research in cognitive psychology and in various subject didactics in subsequent decades. Flavell was a pioneer in differentiating the two basic dimensions of metacognition: metacognitive knowledge vs. metacognitive monitoring and self-regulation: the latter can be subsumed in metacognitive strategies. According to Hasselhorn [5] (p. 37), despite numerous models of metacognition being presented in the following decades, the initial distinction remains fundamental for this area of research. In this sense, metacognition is concerned with (i) declarative knowledge regarding one’s cognitive processes and products and (ii) executive control over these processes.

Metacognition is now considered to play an essential function in learning, particularly in cognitive (learning) processes involving reading and/or writing. More specifically, argumentative writing about complex and controversial topics based on multiple sources requires challenging skills, including strategic thinking, which involves making “metacognitive judgments regarding comprehension quality and adequacy of task performance” [6] (p. 1f.), [7,8]. In these complex tasks, the collation of material from different sources provides the basis for text production because the information from these different sources must be analyzed in relation to each other and used in independent argumentation [9].

List and Alexander [6] (p. 2) highlight that “[d]espite the need for students to demonstrate sophisticated and erudite strategy use when learning from multiple texts, relatively few studies have examined the nature of such strategy use during task performance”. This also applies to empirical investigations and our understanding of the metacognitive experiences or the executive processes of control that are involved. The focus of this study is therefore to address this desideratum [7], and this study aims to contribute to providing insights into students’ strategy use.

The work that is subsequently presented is part of an interdisciplinary research project on argumentative writing based on multiple sources in the geography classroom (SpiGU: “Language Sensitive Teaching and Learning in Inclusive Geography Classes: Support Formats for Material-based Argumentative Writing”, a collaboration between the didactics of geography and the German language). As part of the project, nineteen seventh-grade students from a German secondary school were asked to present their own argument-based position on a conflict regarding the use of space in their city in the form of a written text and to argue for this position after the considering various sources, such as maps, tables, graphics, and newspaper articles. The data collected included both process data (think-aloud protocols on the reading and the writing processes) and product data (written argumentations). In a sub-study, we were interested in whether the quality of the resulting texts is related to the use of metacognitive strategies by the students. In this regard, which metacognitive actions students performed while reading or viewing materials and, subsequently, while writing were derived from the think-aloud protocols. Thus, the guiding research questions for this study were as follows:

− Which metacognitive strategies do students use in argumentative writing based on multiple sources?
− To what extent does the use of metacognitive strategies in argumentative material-based writing have a positive impact on the quality of students’ writing products?

Furthermore, we did not inquire about declarative metacognitive knowledge, but aimed to infer this knowledge by observing the use of metacognitive strategies. To approach the students’ thinking processes, we developed a theory-based model of metacognition in argumentative writing using multiple sources and used the categories derived from this model to evaluate the think-aloud protocols.

In the following work, we first briefly address the discourse on general theories of metacognition (Section 1) and then review the research on metacognition in reading and writing processes (Section 1.2). We then introduce our model of metacognition in material-based argumentative writing (Section 1.3), and, building on this, we present the methodological design of the sub-study in focus (Section 2). Finally, Section 3 presents the results related to the two research questions posed in detail. We close with a short summary and discussion of the findings (Section 4 and Section 5).

### 1.1. Facilitation of Learning Processes through Metacognition

Flavell, who drew attention to the fact that metacognition is something that has to be developed during childhood or school years, understands metacognition as a complex structure in which the two basic dimensions of metacognition are mutually intertwined [4]. The two dimensions are metacognitive knowledge and metacognitive monitoring and self-regulation. Flavell also speaks of experience when referring to the second dimension, and in the following work, we use the summarizing term metacognitive strategies for this purpose.

The close relationship between knowledge and strategies is the most evident in the fact that metacognitive knowledge can come into play within the context of a person’s metacognitive experiences. Personal metacognitive knowledge (knowledge about how to cognitively solve a particular type of problem) may constitute part of the content of a metacognitive experience (i.e., part of the actual cognitive control during a problem-solving process) and then become conscious within that process. Therefore, “metacognitive knowledge and metacognitive experiences form partially overlapping sets” [4] (p. 908). Conversely, meta-cognitive experiences can modify and expand metacognitive knowledge and thus have an impact on cognitive goals and actions.

Following reviews of models of metacognition published in the 1970s, 1980s, and early 1990s, Hasselhorn proposes a classification scheme of the major subcategories of metacognition, which contains five main subcategories [5] (p. 42):

(1) Systemic knowledge: knowledge about one’s own cognitive system and its functioning and knowledge about learning requirements and about strategies;
(2) Epistemic knowledge: knowledge about one’s own current memory states and readiness to learn and about the contents, limitations, and uses of one’s own knowledge;
(3) Executive processes of control: concerning the planning, monitoring, and control of one’s own learning processes;
(4) Sensitivity to the possibilities of cognitive activity: experiential knowledge and intuition;
(5) Metacognitive experiences regarding one’s own cognitive activity: conscious cognitive experiences and conscious affective states.

Hasselhorn [5] (p. 38) emphasizes that one difficulty of metacognition research is to avoid confusing metacognition with other cognitive phenomena and concepts such as motivation or strategies and to differentiate them from each other in the context of empirical studies. An example of this would be, in the area of reading, to compare the cognitive strategy of using a text marker while reading a scientific text with the metacognitive reading strategy of planning, controlling, and evaluating this use [10] (p. 236; see also below).

With regard to certain complex cognitive tasks, metacognition causes the cognitive processes, which are controlled by metacognition in a productive way, to become self-regulatory and autonomous. This means that in the context of teaching, the support and guidance of cognitive processes from the outside becomes less relevant. Instead, the focus shifts to the question of how metacognition can be taught, modeled and structured when acquiring specific cognitive processes. Recently, metacognition has been the focus of research on cognitive reading and writing processes for this precise reason [11,12].

However, no studies have focused on the importance of metacognition during argumentative material-based writing in geography lessons, so an essential research gap can be addressed by the study presented here. In doing so, we build on existing studies on metacognition in reading instructional materials (including texts, graphics, maps, pictures) and in writing as well as in geographical contexts. These are presented in the following sections.

### 1.2. Studies on Metacognition in Reading and Writing Processes

Previous studies have focused on either reading or writing processes, with a particular focus on metacognition in reading individual texts and single sources. As early as the late 1990s, Baker [13] provided a synthesis of the former research on metacognition and comprehension monitoring among adult readers, which included college students. This work emphasized that students who have a great level of reading expertise and who are more successful in their studies “seem to have greater awareness and control of their own cognitive activities while reading” [13] (p. 33), [14]. Better readers are more likely to make an effort to overcome comprehension difficulties when they notice them [15,16]. However, even when adults and students more fully evaluate and regulate the process of their efforts to understand what they read, there are often still developmental needs. Basically, in the field of reading, metacognition has to be developed over the course of adolescence. Thus, “the evidence of age-related changes in the metacognitive skills of older adolescent and adult readers is intriguing” [16] (p. 34).

Similarly, van Kraayenoord [17] provides a summary of the extensive literature on the role of metacognition in reading comprehension and discusses recent studies from Italy, Israel, and Australia. Van Kraayenoord concludes “that it is clear that with development students become more aware of their own thinking about themselves, the tasks and the strategies that are useful for reading and that good comprehenders are more aware than poor comprehenders” [17] (p. 292). With respect to the factors that best account for reading comprehension, van Kraayenoord [17] (p. 284) refers, among others, to a study by Meneghetti et al. [18] that highlighted that the key complex metacognitive skills involve, among other things, the differentiation of relevant and non-relevant information as well as the adaptation of reading strategies to the specific text type.

In terms of the metacognitive monitoring of one’s own reading comprehension process, these studies indicate that monitoring can be influenced by “the students’ prior knowledge, perceptions of texts as accurate and coherent, knowledge of language, reluctance to admit to comprehension problems, and a propensity to view reading as a decoding activity, especially in young readers, those with difficulties in comprehension and those with learning disabilities” [18] (292f.). At the same time, according to Kraayenoord’s conclusions, on the one hand, metacognitive knowledge is primarily domain-specific (you can have it in a particular area and not another). On the other hand, better readers have more strategies for controlling their reading comprehension and can adapt their control strategies to a particular text better than poorer readers [19]. Consequently, this implies that reading instruction and support should also integrate the teaching of strategies that help to monitor and control the comprehension process [19] (p. 294).

Ahmadi et al. focus on the context of educational processes and highlight the im-portance of metacognitive reading strategy awareness in reading comprehension. The study differentiates cognitive reading strategies such as “guessing from the context, using a dictionary, writing down, imagery, activating background information, summarizing, using linguistic clues, using text markers […]” [10] (p. 236) from metacognitive reading strategies. With respect to the latter, Ahmadi et al., following Jacobs and Paris [20], further differentiate these categories into three main classes [10] (p. 237f.):

(1) Planning: Choosing appropriate cognitive reading strategies and sequencing them appropriately in response to a particular reading task; on the level of forethought, planning is generally the process of thinking about and organizing the activities that are essential for achieving a particular reading goal or for successfully completing a reading task.
(2) Monitoring: Analyzing the processes involved in one’s own reading and comprehension and assessing them in terms of their effectiveness and efficiency. In this sense, monitoring is a control tool that prepares the basis for the evaluation of one’s own reading and comprehension performance.
(3) Evaluation: During evaluation, the ongoing or completed reading and comprehension process is estimated and judged. The (self-imposed) reading goal or the higher strategic plans are compared with the actual implementation and the associated accomplishments.

In general, according to Ahmadi et al. [10] (p. 240), “[c]ognitive [reading] strategies are important to perform a task, while metacognitive reading strategy awareness is necessary to recognize how the task has been performed”. Furthermore, Ahmadi et al. stress that, overall, the research findings suggest that while the explicit teaching of cognitive reading strategies can support small, short-term developments in reading performance, fostering metacognitive strategies promotes more stable, long-term comprehension [10] (p. 241). Thus, the results indicate that the use of metacognitive strategies has a positive impact on learning outcomes. The question is to what extent this also applies to reading and writing processes when learning through the use of multiple sources. Three examples of studies that address this topic area are presented below.

Goldman et al. [21] concentrated on the reception (reading, comprehending, and learning) of multiple sources from the Internet and compared the processing of 10 better learners to 11 poor learners when performing a web-based exploratory task. They found that “better learners engaged in more sense-making, self-explanation, and comprehension-monitoring processes on reliable sites as compared with unreliable sites, and did so by a larger margin than did poorer learners. Better learners also performed more goal-directed navigation than poorer learners” (p. 356). The outcome of this study indicated that understanding the contexts extracted from multiple sources is a dynamic process that involves the interaction of “sense-making, monitoring, and evaluation processes, all of which promote strategic reading” [21] (p. 356).

Combining research on reading and writing using multiple sources, Anmarkrud et al. [22] (p. 64) used a “think-aloud methodology to examine the strategic processing of 51 Norwegian undergraduates reading about an unfamiliar scientific issue in multiple conflicting documents.” They found that “students’ strategic processing during reading was related to their written argumentation, with evaluating, monitoring, and cross-document linking positively related to argumentative reasoning about the scientific issue” [22] (p. 64). Furthermore, among other things, Anmarkrud et al. concluded that “the more carefully readers monitor their emerging understanding of the issue, and the more actively they regulate their approach to the task in response to the results of the monitoring process, the more likely it is that they succeed in bridging different perspectives and piecing together an integrated understanding of the issue” [22] (p. 74).

As a final example, we refer to List and Alexander [6], who investigated undergraduate students’ strategy use when learning about a complex and controversial topic using information from multiple sources. In this experimental study, 71 students from the United States were asked to identify information in texts that was easy or difficult to understand. It was found that the use of strategies related to multiple texts improved task performance.

Overall, current research suggests that metacognition can have an important influence on the comprehension process and processing success, not only when reading simple texts, but also, and especially, when reading (and writing) using multiple sources. The latter is the typical way of writing in geography lessons. Studies on geography education show the positive influence of metacognition on geographical learning. The positive influence of metacognitive strategies on the quantity and quality of the formulation of geographical causal relationships was proven by Heuzeroth and Budke [23]. The metacognitive strategies provided to the students also had a great influence on the correctness of the causal relationships that they formed. It was found that the higher the students’ ability for linguistic complexity, the more likely they were to formulate geographical causal structures correctly with regard to content. Furthermore, Setiawan et al. [24] demonstrated a positive effect of students’ metacognitive skills and learning outcomes in geography education.

### 1.3. A Model of Metacognition in Argumentative Writing Based on Multiple Sources

**Figure 1:**
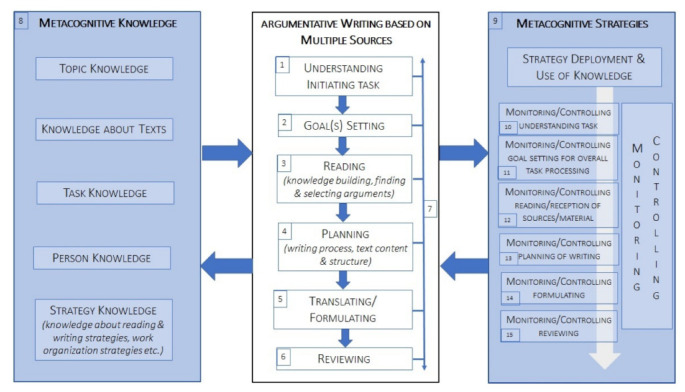
Model of metacognition in argumentative writing based on multiple sources (own elaboration).

The theoretical and empirical work on metacognition in learning processes, particularly in reading and writing, that were reviewed in the previous section forms the theoretical basis for the model of metacognition in material-based argumentative writing that we propose (see Figure 1 below). The conceptual anchor point for this model is still the fundamental work of Flavell, which we relate to the cognitive processes involved in writing using multiple sources, among other things, in alignment to the pertinent writing model of Hayes and Flower [25]. In the following section, we first summarize the basic steps of material-based writing, including the preceding (potentially circularly intertwined) components of multiple-document reading [26,27,28]. We then show the model with its various metacognitive dimensions, which we subsequently explain in more detail.

The initial task is the initiation of argumentative writing using multiple sources and usually represents the basis from which further activities are undertaken. Strategic writing starts with understanding the task (see box 1 in Figure 1) and setting goals in relation to the individual stages of the work process (box 2), which, in this case, involves setting goals with respect to the reading and writing process as well as to the final result (i.e., the desired function and effect of the text).

The reading and comprehension of multiple texts and the reception of distinct materials such as maps, tables, graphics, and newspaper articles are components of a knowledge-building process that involves finding and selecting appropriate arguments (box 3) when developing a piece of argumentative writing.

Building on the reception of the materials, the planning step includes the planning of further procedures with regard to previously defined goals (box 4). This step contains the planning of the text structure and considerations about the text content, with a particular focus on the integration of previously collected arguments identified from the source material as well as one’s own arguments and subsequently determining the order and hierarchy of those arguments. During planning, it is also possible to consider how to refute individual arguments and how to present one’s own position convincingly.

The translating (or formulating) step is concerned with writing and composing (box 5). Finally, reviewing includes reading and revising your own text (box 6). The aim of the final stage is to evaluate the extent to which the original objectives of the task have been met.

Even if task processing can be imagined as a linear process, it is understood to be recursive, as over the course of processing, it is always possible to jump back to an earlier step. This recursive process is indicated by the two-sided arrow on the right-hand side of the section for the modulation of argumentative writing based on multiple sources (box 7).

As outlined in the previous chapter, the two fundamental dimensions of metacognition, metacognitive knowledge and metacognitive strategies, are mutually intertwined. Consequently, we visualized them as two dimensions that, when a person performs metacognitive processes, flank the process of working on the material-based writing task but, at the same time, are to be perceived as belonging together (marked by the uniform grey colouring: box 8 and 9).

The model includes five subcategories of metacognitive knowledge (box 8). In addition to external resources in the form of materials, writers use their knowledge of the subject, of the nature and structure of argumentative texts, and of reading and writing strategies. Personal knowledge, according to Flavell [4] (p. 907), includes knowledge about one’s own cognitive learning processes, their intra- and interindividual peculiarities, and their universal aspects. The category “Task knowledge” refers to one’s own estimation with regard to knowledge about the task type [4] (p. 907). Strategy knowledge is knowledge regarding which strategy is the most effective for achieving a certain goal [4] (p. 907). As Flavell’s stated, these individual knowledge areas interact with each other.

In box 9, we modelled the dimensions of metacognitive strategies: When strategies are deployed and knowledge is used, a person can become aware of their own knowledge as well as their thinking strategies and can consciously monitor and control them. Modelling this strategy deployment and use of knowledge with respect to the argumentative writing process using multiple sources, we follow Hasselhorn’s [5] (p. 42) classification scheme of the executive processes of control (see above, Section 2). As such, for each basic step of material-based writing, we distinguish the respective instances of metacognitive monitoring and control. Thus, metacognitive strategies come into play in the first step of understanding and initiating the task, which occurs when the person monitors or controls this step (box 10) and for each step of task processing (box 11 to 15). (Note the difference between the category planning the writing process in the context of task processing (box 4) and monitoring/control of that planning as a metacognitive strategy (box 13). Many classifications of metacognitive strategies also list planning (as does Hasselhorn, ibid.). In order to be able to conceptually distinguish the process of *cognitive task processing* for planning the writing task and the *metacognitive processes* involved in it, we distinguish between planning processes that also potentially take place unconsciously (*cognitive*) and the conscious monitoring of these planning processes (*metacognitive*).

In the following chapter, we outline how we used the deductive category framework in order to assess the data collected in our metacognition sub-study in more detail.

## 2. Methods

### 2.1. Participants

The presented study was undertaken with 8th-grade students from a secondary school (a *Gesamtschule* in a socially deprived area) in North Rhine–Westphalia (NRW, Germany) in February 2020. The sample size was 19 students (*n* = 19 students, male: *n* = 10; female: *n* = 9) aged between 13 and 15.5 years old. The majority of the students were multilingual, with German being their second language. However, all of the students were born in Germany. A total of three students had been diagnosed with special needs in learning (SLD), with an emotional or social disorder (ED), or a combination of both SLD and ED (SLD/ED). (We refer to students with special needs as follows: SLD = Student with a Specific Learning Disability, ED = Student with an Emotional or Social Disorder, SLD/ED = Student with both types of special needs.)

### 2.2. Procedure

All of the students were asked to review several sources regarding a land use conflict in their city and to write a statement about the conflict. The assignment addresses a socially relevant issue, something that is typical for geography classes at this level. The intention was to present the land-use conflict from different perspectives. For this reason, the materials that were used came from diverse sources and mainly included maps, excerpts from a petition text, and authentic newspaper articles, photos, etc.

### 2.3. Instruments

In the writing and reading process, students were asked to use the think-aloud method to verbalise their actions and thoughts. In order to address the first research question (*Which metacognitive strategies do students use in argumentative writing based on multiple sources?*), we analyzed the collected process data, namely the think-aloud protocols of the reading and the writing processes, using the qualitative content analysis method [29].

### 2.4. Data Analysis

For the data analysis, we used deductively developed categories based on our model of metacognition in argumentative writing using multiple sources (see Figure 1). An overview for the entire main and subcategories of the analysis can be found in the appendix of this article. The analysis guide focused on the empirical reconstruction of the metacognitive strategies that the students executed during the steps of the task (see Figure 1: boxes 1 to 6). According to the model, the coding encompassed six main categories that focussed on the expected metacognitive strategies in the respective phases. Thus, we included “monitoring or controlling the understanding of the initiating task” (cf. box 10), “monitoring or controlling the goal setting for overall task processing “(cf. box 11), and the four other strategies in this section (box 12 to 15). These expected metacognitive strategies, which were tightly bound to a specific process step, were further differentiated into several subtypes. In other words, the subtypes represent the more specific kinds of metacognitive strategies that the participants used during the individual phases of the writing process. We exemplify this procedure in the following illustration (see Table 1) by presenting the subtypes of the expected metacognitive strategies belonging to the first process step, (1) *Understanding Initiating Task.* We do not present the subtypes in detail for the other process steps and instead focus on the metacognitive strategy as the main category. (See Appendix A for an overview of the entire code tree with all of the main categories and subcategories).

The second research question (*To what extent does the use of metacognitive strategies in argumentative material-based writing have a positive impact on the quality of the students’ writing products?*) required us to relate the students’ identified metacognitive strategies to the quality of the argumentative text they produced. These written statements were examined using a theory-based catalog of criteria, which had been developed in an interdisciplinary manner during the SpiGU project [9]. The catalog identifies the following areas of analysis:

(1) Argumentative design of the text, based on Feilke’s theory of text procedures [26,30,31];
(2) Linguistic and structural organization of the text (lexis and grammar);
(3) Reference to material;
(4) Quality of the reasoning.

The approach of Toulmin [32] was used to measure the structural quality of an argumentative text. When measuring the content quality, geography-related content criteria were defined and put into operation [9]. Overall, the examined quality scores of the students’ written products were correlated with the scores of the think-aloud protocols.

Before we introduce the results of both research questions, a final point regarding the methodological design to be highlighted is that the use of the think-aloud protocols needs to be critically reflected upon in this approach. This is because in principle, the request to think aloud can trigger metacognitive processes that would not be realized without its use. It is also sometimes difficult to differentiate between a metacognitive activity and a mere utterance of thoughts. This problem is fundamental and ultimately unresolvable since access to metacognitive activities can only be reconstructed through verbalization.

## 3. Results

**Figure 2:**
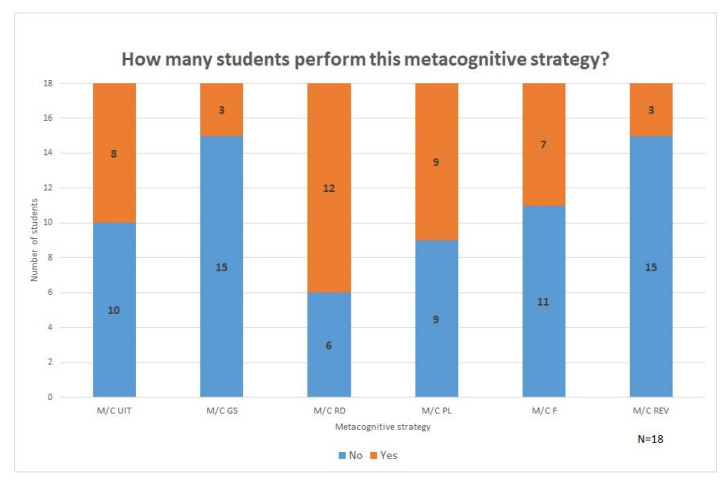
Proportion of students using each metacognitive strategy (own elaboration): M/C UIT = Monitoring/Controlling Understanding Initiating Task, M/C GS = Monitoring/Controlling Goal(s) Setting, M/C RD = Monitoring/Controlling Reading, M/C PL = Monitoring/Controlling Planning, M/C F = Monitoring/Controlling Formulating, M/C REV = Monitoring/Controlling Reviewing.

The results are presented in two sections: Section 3.1 focuses on the first research question and shows how, for each main category of metacognitive strategies (see Figure 2, Section 3.1), which ones, according to our model (Figure 1, Section 1.3), can be applied in the processing of the material-based argumentative writing tasks and which ones the surveyed students *actually* performed. Subsequently, Section 3.2 addresses the second research question and specifies the extent to which the use of metacognitive strategies and the quality of the task objective, the written argumentative texts, were correlated.

### 3.1. Results concerning Metacognitive Strategies Used by Students

In the following section, we illustrate the evaluation results by displaying them for each of the six defined metacognitive strategies. For this purpose, we include the results obtained for the differentiated subtypes in order to provide a more in-depth perspective. Figure 2 provides an overview of how many students used a particular metacognitive strategy during the working process (values are shown in percentages for a total number of 18 students).

Figure 2 shows the extent to which different metacognitive strategies were used and shows that, on average, they were used in less than half of the analyzed students’ think-aloud protocols. Initial impressions suggest that overall, students *do* make use of metacognitive strategies. However, when looking at the results in detail and comparing the usage of the different strategies, the variations between their use are large.

A limited number of strategies were used by the students, which is particularly evident in the two categories *Monitoring/Controlling Goal(s) Setting* and *Monitoring/Controlling Reviewing*: here, only 17% of all students (three students, one with ED) used metacognitive activities (i.e., metacognitive monitoring and self-regulation). The greatest amount of strategy use was observed in the category *Monitoring/Controlling Reading*, where metacognitive activities were identified in 67% (twelve students, one with SLD/ED) of the students’ think-aloud protocols. Overall, the results suggest that the use of metacognitive strategies is common. However, it is appropriate to look into the results for each strategy to gain further insight.

#### 3.1.1. Metacognitive Strategy Use: Monitoring/Controlling Understanding of the Initiating Task (M/C UIT)

For *M/C UIT*, 13 metacognitive verbalizations were identified from the think-aloud protocols of 8 students (44%) from the total of 18 students. The quantitative distribution of the five related subtypes (see Table 1) showed the following results:

Only 6% of the students (one student) made use of the strategy concerned with the first subcategory, “Thinking about the action required to complete the task”. Additionally, 11% (two students) could be assigned to the fourth subcategory concerning the awareness that the material needs to be read/evaluated, and 6% (one student) could be assigned to the fifth subtype regarding the insight that information and arguments needed to be extracted from the material. Most of the metacognitive activities that were identified focused on the second and third subcategory with regard to the task of developing one’s own opinion and the requirement that the opinion needed to be justified.

According to this connection, only 22% of the students (four students, one with ED) explicitly stated that they were aware of the requirements of the tasks. They used metacognitive strategies to understand the expectations of the task and to derive necessary actions. Four students recognized that they had to formulate their own opinion on the topic. For example, S19 (the students in our sample were numbered for the purposes of anonymization, and individual students are referred to with an abbreviation: student 19 = S19) stated the following: *“Now I’m supposed to write what I´m thinking about it, I´m supposed to write whether I’m for it or against it”*. Three students (17%, one with SLD/ED) addressed an advanced aspect of the task, which was to justify their own opinion. S1’s utterance “*I´ve justified my opinion*” indicates that he understood that justification of their opinion was an essential task requirement, in comparison to S20, who showed that he was uncertain as to whether a justification of his opinion was required by asking the instructor “*Are we supposed to justify or not? I mean the text?*” Furthermore, it is interesting to note that S19 misjudges the task by stating that “*The task does not say that we´re supposed to justify this. That’s why I don’t justify it*” despite previously correctly summarizing the requirement of opinion development. When considering other subtasks in the complex assignment (i.e., evaluating the material and extracting/writing arguments), the amount of metacognitive reflection was still minor, with only 6% of the students (one student) explicitly identifying the components of the different tasks. A positive example was S11, who noted the following: “*So, I´m collecting counterarguments and arguments in favor of it*”.

The results for *M/C UIT* showed that no student accessed *all of the* metacognitive strategies when reflecting on the requirements of the task and that just eight students (44%) reflected upon some of the tasks. Ten students (56%) did not reflect on the assignment at all. These results suggest that the students did not transfer any, or transferred very few, of the various requirements of the task into their mental working memory. As such, it is likely that the students had an incomplete or unclear understanding of the assignment. It is also possible that they do not understand the different aspects of the task, either.

#### 3.1.2. Metacognitive Strategy Use: Monitoring/Controlling of the Goal Setting for Overall Task Processing (M/C GS)

With respect to setting global goals, we defined the leading metacognitive strategy as follows: The student sets goals for the processing of the overall task, including for its involved subtasks and structures.

From a metacognitive perspective, this category is about (a) identifying the subgoals of the global task and (b) thinking about how to sequence and organize one’s own working steps to achieve these subgoals. The subgoals are characterized by the fact that the land-use conflict presented by the material as well as the associated positions of the actors are to be mentioned in one’s own text product. Secondly, the text to be written should pursue a specific text function (statement) that takes the associated text-type conventions into account.

As previously mentioned (see Section 3.1, Figure 2), the category *M/C GS* contributes to a small proportion of the metacognitive activity identified in the data. Only four metacognitive verbalizations, carried out by 17% of the students (three students), were assigned to the main category, and, more specifically, the metacognitive strategies of two subtypes were not performed by any student. That is, none of the 18 students reflected on the goals of (a) explaining the conflict and (b) outlining its associated multiperspectivity in their text. With respect to the third subcategory, “Setting the goal of realizing the intended text function (letter/statement) by writing the argumentative text in an addressee and goal-oriented manner”, only one student (6%) executed the expected metacognitive activity. That is, S20 stated “*I will now write down my statement*” (indicating that he was about to write his text as the next step in the working process) as well as named the specific text function (statement). S20 also performed metacognitive strategy use through consideration of the sequence of his retrospective and upcoming actions (“*Now I’ve gone through all the sheets, and now I’ll look at the task again.”).* Similarly, another two students (17%) defined an order for their next working steps by setting interim goals. This included first rereading the assignment after receiving the material and then determining an immediate reading order for the material. Metacognitive strategy use was illustrated by the verbalizations of S16: “I´ll now look at the tasks again.”, and S17: “*This is a long text. I might read through it later*”.

Summarizing the observations for *M/C GS* and thus the setting of task-related (interim) goals, we found that very few students reflected on the essential goals of the task metacognitively. Most of the students did not think about or plan the sequence of their actions. However, the few students who were metacognitively active (e.g., S20) showed strategy use in more than one subcategory. Furthermore, those who did not use metacognitive strategies in *M/C UIT to understand the assignment* did not show strategy use in *M/C GS* either, with one exception (S17). Consequently, the metacognitive strategies implemented for task comprehension therefore appear to form the basis of metacognitive strategies for other areas of material-based argumentation, although, when comparing the eight students who used metacognitive strategies in *M/C UIT*, only two of them (S16 and S20) also used these strategies in *M/C GS.*

#### 3.1.3. Metacognitive Strategy Use: Monitoring/Controlling the Reading/Reception of the Material (M/C RD)

The main category, *M/C RD,* deals with strategy use that is oriented towards monitoring and controlling the reception of task-related material (texts, maps, statistics, pictures, etc.). It includes seven subcategories of metacognitive strategies (see Appendix A), and the overall leading metacognitive activity is as follows: *The student reflects on the steps involved in reading/evaluating each material.*

In these steps, students should think about the use of possible reading strategies in alignment with the individual materials provided and the reading modes that are specific to them. This includes, for example, taking notes, establishing an internal reading order for texts, or reading the legend for maps/statistics first. The task-based reception of material also involves selecting evaluation strategies to extract relevant arguments from the sources, linking them, and establishing a hierarchy for the selected information and arguments with regard to one’s own text.

Furthermore, this step involves competence in evaluating material with regard to its relevance to master the task as well as to assess the credibility of the information in the material provided. Reflecting on the linguistic composition of the material is also part of *M/C RD*. For example, this reflection process includes reflection on direct and indirect meanings, irony, and other rhetorical features. Lastly, a student can explicitly name the problems he or she identifies during the reading process and, by doing so, filters the challenges he or she encounters.

Overall, the use of metacognitive strategies related to the main category by the sampled students was identified in 67% (twelve students) of the 18 think-aloud protocols. Focusing on the strategy where the fewest students were metacognitively active and the strategy where the most students were active shows that none of the students (0%) reflected on the importance of comparing the relevant information from the material, hierarchizing it, and checking it for contradictions and repetitions. In contrast, more students (seven students, 39%) verbalized problems that they encountered while reading. For example, several students reported difficulties in understanding information, such as S13: “*So, they’ve started talking about rail, parking, and stuff, but I’ve read through it a couple of times now, I don’t really understand what it’s about. Why they´ve started talking about streetcars and parking*”. The students were therefore partially aware of their comprehension problems, but hardly any solutions were derived from this awareness. S13 attempted the strategy of reading the text several times, but this did not solve the difficulties of his understanding.

A qualitative in-depth analysis of the use of different metacognitive strategies revealed that one student (6%) considered whether the information that had been received should be written down, as S12 asked the instructor the following: “*Should I write anything down about this? So, I can’t write anything down yet?* (Instructor: Asking whether S12 means note-taking?) *No, I can’t write anything down here*.” S12, by consulting the instructor, thought about the extraction of information relevant for the fulfilment of the task and thereby considered the evaluation strategy of writing information down. However, S12 did not seem to trust his own competence of making this decision alone, which is why he requested advice from the instructor.

Concerning the use of reading strategies, there were only two students (11%), S7 and S20, who showed monitoring activity concerning their procedure for reading the material. S7, after reading all of the materials, decided to review the materials again in preparation for planning and writing his own text, stating the following: “*I´ll look through all the pages again*”. This statement can be interpreted to mean that S7 wanted to review the different materials against the background of the overall task context in order to obtain an overview of the conflict and the information to be discussed.

S20 reported that he recognized graphic elements when looking at a map and actively decided to use the strategy of initially exploring the different markings on the map: *“Here I see Cologne. And there are various markings on it, at which I’m going to take a closer look now”.*

Another student commented on the typographic design and subject-related terms in the material at hand; from the pictorial design of a material (in which two actors of the conflict and their corresponding speech bubbles were depicted), S8 inferred that there was a conflict between the conversation participants: “*One is against it, one is for it. And I think that they´re having an argument, as you can see here, because there are a lot of speech bubbles or thought bubbles (…)*”. In addition, S8 also referred to the typographical highlighting of lexical entities in the speech bubbles by explaining that “*(…) there are a lot of speech bubbles or thought bubbles, even capitalized once. So a little attentive, I think, and underlined*”. Thus, the student expressed the assumption that these typographical highlights were being used to draw the reader’s attention to the respective lexical units. S8 therefore executed metacognitive reflection on the form–function relationship of the linguistic entities in the material at hand.

Other evaluation actions were focused on either the credibility of the material or the students’ subjective interest in it. Such evaluations of the material occurred in three of the students’ protocols (17%). An example of this was S2, who recognized the computer-based editing of a visual material (image material) by stating the following: “*Here I see a picture, M8. This looks designed with Photoshop*”. The comment indicates that S2 metacognitively reflected on the artificiality of the specific material and verbalized his subjective perceived impression of it.

The last subcategory concerns the evaluation of material in terms of its relevance to the fulfillment of the task. Although 22% of the students (four students) were found to execute this metacognitive activity, none of them explicitly emphasized that a material and its information and arguments were useful. In contrast, two students did express the opinion that they did not consider a particular material to be helpful. For example, after reading all of the information provided, S13 evaluated the material as a whole by reflecting on its usefulness: *“So I’ve read through the whole thing now and I’ve gotten nothing from the most, so in short, none of it is seen as an argument. The only argument, in short, my arguments are that I’m against it”.* Considering the fact that S13 expressed comprehension problems several times during the reception of the material (see, e.g., the utterance of S13 above or “*I don’t think anything about the next sheet, it just says how many members the football club has and where they play in the German league, that tells me nothing actually*”.), it is not surprising that S13 came to the conclusion that he had difficulties extracting relevant information from the material or evaluating it as useful for his own text, as he did not understand a large part of it.

For *M/C RD*, more than half of the students (67%) demonstrated metacognitive activities, but most of them expressed comprehension problems. The students did not attempt to solve these problems, and they were, instead, accepted by the students, and the reception of the material was continued. Furthermore, it was evident that the students did not have sufficient reading and evaluation strategies that they could use for the task-related processing of the material or to control and monitor their reception process. Finally, although some students carried out evaluation activities when reviewing the material, they did not focus on evaluating the information in a different way or on extracting useful information accordingly. The material was not reflected on based on the usefulness of the information it contained but was instead considered in terms of how the material was formally designed.

#### 3.1.4. Metacognitive Strategy Use: Monitoring/Controlling the Planning of Writing (M/C PL)

As a fundamental part of the working process in argumentative writing using multiple sources, the following metacognitive action was defined for describing and summarizing the monitoring and controlling activities during the planning phase: *The student reflects on the planning/structuring of the writing process and on the composition and structure of the text product.*

This metacognitive action can take the form of four different metacognitive strategy subtypes (see Appendix A). It can be expressed through the student’s considerations of the order in which they proceed with the writing of the text or the text-production steps they would like to include. For example, the student may decide to first collect arguments from the material and then to integrate them into their own text. A metacognitive planning strategy of this kind can also take the form of the student explicitly communicating that they will return to the material during the formulation process in order to check the source or the material content that they will use in the written text, as the metacognitive monitoring of planning is also reflected on through the process of thinking about the structural and/or the content level of the text to be written and the interdependence of these through the use of appropriate strategies. Lastly, a student can explicitly name the problems they identify during the planning process and, by doing so, can filter the challenges they encounter during the planning phase through metacognitive monitoring.

We identified such occurrences of *M/C PL* in the think-aloud protocols of nine students (50%). One student (6%) confirmed his planning with regard to the structural composition of the own text, and 33% of the students (six students) planned the order of the writing and the related steps when writing the text.

Taking a qualitative perspective on the use of the different metacognitive strategies reveals that S11 was the only student who reflected on the structural organization of the own text. By using the statement *“How should I start? What I´m thinking when I´m writing? Simply my own opinion”*, S11 gained insight into a moment of text planning. In this moment of planning, the student thought about the order in which he should proceed in writing the text and, in the course of this, with what aspect he should begin. S11 decided to formulate his own opinion first. Through comparison with the text product, it was apparent that S11 implemented this plan; the first sentence of the text reads “*I am definitely against the construction of more sports fields*”.

In addition to S11′s planning, three students (17%) verbalized that they had a problem in the context of planning. An example of this was a statement made by S12: *“I don’t want to write a whole novel now, as I usually do in Geography lessons, because then I digress from the topic and that would no longer be appropriate for the task requirement”.*

It seems evident that S12 is aware of a challenging aspect (personal to him) when he is faced with the task of writing a text. S12 reflected that he wanted to avoid digressing from the topic of the assignment. In order to counteract this self-identified difficulty, S12 metacognitively searched for a solution and came to the conclusion that he could achieve this through limiting the length of the text.

With regard to metacognitive strategy use concerning content planning, 28% of the students (five students) showed activities that corresponded to this category. One example was S11, who reported that he explicitly wanted to extract pro-arguments and counterarguments from the material: “*So, I´m collecting counterarguments and arguments in favour of it*”. Another example was S20, who not only engaged in a higher frequency of content planning compared to the other students (six activities in total), but verbally summarized, differentiated, and detailed the content he wanted to use in the text in advance: *“I see no place where you could do that, so I recognize no place where no green is. But there are certainly still many places where you could build a new park. And I think it´s logical, if then the 1.FC. Cologne builds its own place, these training grounds, and pay it from its own money and then maybe donates a little money to the city, so that the city can in turn build a new park somewhere. I’ll write that down again now”.*

Comparing S20’s planning comments with his text product showed that he incorporated the pre-planned content into the text: “I am also in favor of the 1. FC Cologne paying for its training ground itself and donating money to the city so that it can build a new park at another location or expand an old park”.

Finally, 33% of the students (six students) focused on naming the next step in the sequence of their working process based on their current work status. An example was S13 who, at the point where he had completed the reception process and was moving on to the phases of planning and formulating, explained: “*I’ll now write down what I’ve thought throughout the conversation and will also read through the sheets now and then*”.

Summarizing the observations for *M/C PL,* we found that 50% of the students reflected on the planning process as well as on the text to be written metacognitively. However, we observed that the students’ monitoring and controlling activities concerning their metacognitive planning lacked depth of thinking. The students mostly operate superficially by naming their next working steps, for example, but not describing them further with regard to their execution. S20 was an exception to this, as he described what he wanted to integrate in advance. The frequent use of content planning strategies correlated with the quality of S20’s text, as it was the strongest text product of the sample.

#### 3.1.5. Metacognitive Strategy Use: Monitoring/Controlling of the Formulation (M/C F)

The guiding strategy characterizing metacognitive reflection during the formulation phase can be defined as follows: *The student reflects on the written communicative actions and the formulations of the text product.*

The following subcategories can be identified within this strategy: Firstly, monitoring/controlling the formulation can involve metacognitive reflection on the linguistic entities, i.e., the formulations that the students then use in their texts. Thus, the writers reflected on the appropriate use of written argumentative procedures (i.e., the use of *on the one hand...on the other hand* to contrast arguments and information). In addition, during the writing process, students also reflected on whether the language they chose fit the required text type (i.e., the use of linguistic expressions to demonstrate politeness). Furthermore, there is also the metacognitive control of the grammatical and orthographical correctness of the writing as well as the use of appropriate strategies to correct discovered errors and uncertainties (i.e., asking the instructor, looking up a word in the dictionary, etc.). Lastly, as in reading and planning, monitoring the formulation can involve identified problems being explicitly addressed in the writing process.

A total of 18 metacognitive verbalizations carried out by 39% of the students (seven students) were assigned to this category. More specifically, the quantitative distribution of the weakest and strongest subcategories was as follows: No metacognitive reflection (0%) on the formulations in alignment with the targeted text type was observed in any of the student samples. In total, 28% of the students (five students) showed strategy use with regard to thinking about linguistic formulations and argumentative language use.

The qualitative in-depth analysis revealed that one student (6%), S12, made an evaluative comment about his orthography by saying “*Oh God, my spelling*”. This happened while S12 was writing, expressing his immediate awareness that the text was deficient in that regard. Four students (22%) recognized and named difficulties and problems that they perceived during the writing process. Again, an example stems from S12’s protocol. That is, he described his personal perception of the current state of the writing process based on the following statement: “*Oh, oh, writing blockade. What else should I write? Full stop. What should I write? I’m kind of pressed for time right now, and I didn’t want to be pressed for time*”.

The example above shows that the student was searching for further aspects or information to include in the text. However, S12 realized that he could not find any at the current stage and described this circumstance as *blockade*. In addition, he then had the feeling that this blockade was causing a time problem in the formulation process and, consequently, in the completion of the text. In addition to S12, two other students (S2 and S22) also mentioned the problem of not knowing what else to write.

As previously mentioned, the majority of students (five students, 28%) thought about possible formulations and the argumentative language that they could use by searching for formulations in the material. An example of this was S10: “*But I also understand that somehow, because the club has more than, wait I’m looking briefly, (...) 350 players, and in addition there are also all the fans”.*

Another strategy was to turn to the instructor, as S2 did, to ask whether he can write down that he would like to play for the football team in question in his text.

Overall, a small number of students (seven in total) monitored their own formulation process. For these students, it was apparent that they focused on the requirement of introducing further information into the text and, to achieve this, having to search for appropriate formulations. A large part of the students evaluated this as a problem and described it as not knowing what and how to write: “*What else* *should I write? How should I phrase this*?” (S1).

#### 3.1.6. Metacognitive Strategy Use: Monitoring/Controlling the Reviewing (M/C REV)

The final main category *M/C REV* is characterized by various strategies that belong to the following main metacognitive activity: *The student reflects on the steps required for text revision.*

In this respect, we examined the data using the following stages: (see Appendix A) First, we reconstructed whether and how the students reflected on the essential steps of the review process; second, we coded to what extent they checked the content and formulations of their text products and revised them; third, we were interested in whether they confirmed that their written argumentation corresponded to the required text goal/effect specified by the task.

As with *M/C GS*, category *M/C REV* shows that only three students (17%) used revision strategies. Altogether, *no* occurrences were found in the data material that pointed to controlling correspondence between the written argumentation and the required text task, with none of the students having explicitly checked the intended task-related text goal. Since no or very few targets were set in advance (see results on *M/C GS)*, this finding was expected.

However, two students (S9 (ED) and S11) thematized their written texts on the formal level of readability and in terms of content. For example, S9 reassured himself in a conversation with the instructor that a certain passage in the text was readable by asking the following: “*I’m finished. I couldn’t erase it, is that bad?* (Instructor is replying that it is no problem). *Can you read it?* (Instructor is confirming that student can read it)”.

S11 considered the final text with regard to the content level by emphasizing his own position on the conflict based on predominant counter-arguments extracted from the material. In addition, S11 described that he had also integrated the same content he expressed when thinking aloud into the text: “*So now my text. I have actually already explained everything I said before. I think that they contradict themselves all the time and there are too many reasons why you shouldn’t do that*”. One could say that S11 looked at his own text from a bird’s-eye view and reflected on the content components that constituted the written argumentation.

The results for the final category *M/C REV* show that no structured procedures of text revision were identified in the student samples and that hardly any students used metacognitive strategies during their review processes.

### 3.2. Results concerning the Impact of Metacognitive Strategy Use on the Text Quality

**Figure 3:**
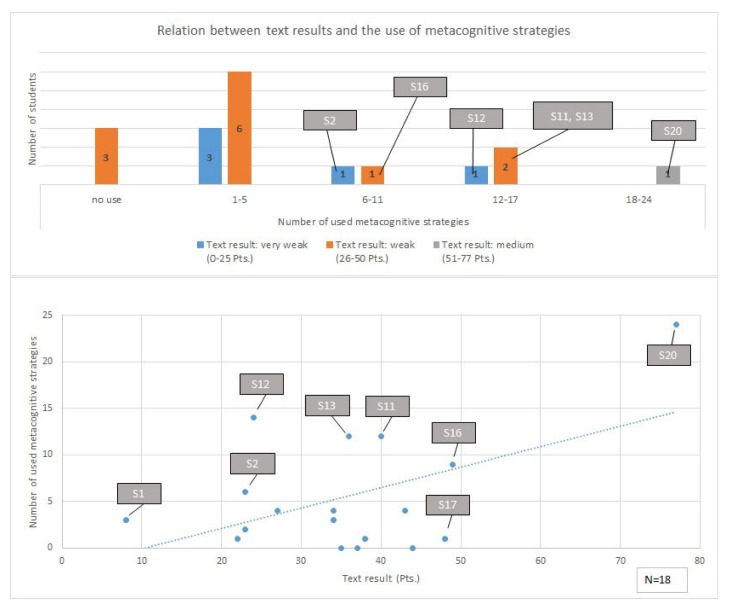
Relationship between the text quality and the use of metacognitive strategies (own elaboration).

In the following section, we first explore the relationship (correlation) between the few metacognitive strategy used by the students and the quality of their argumentative texts (see Figure 3). Following this, we present what and how many occurrences of metacognitive activity can be reconstructed from the think-aloud protocols for each of the 18 students in more detail. Here, we show the percentage of the different metacognitive strategies used by each student.

A series of key points can be identified from these results. The student who wrote the best text (S20) used significantly more metacognitive strategies than their classmates. Most students (12 in total) did not use any or less than six metacognitive strategies and wrote argumentative texts of poor (26–50 Pts.) or very poor (0–25 Pts.) quality. The latter of these observations is supported through further analysis of the empirical results. Concerning the question of material reference (this question was also investigated as part of the SpiGU project), it was clear that the majority of the written texts are deficient in these strategies because the students in particular find it challenging to filter relevant information from the material and, as a result, they integrate very few of the materials into their own argumentative texts (an average of 1.9 materials) [24] (p. 28). Although the students read all ten materials, they lacked a strategic approach to filtering the central information and arguments from the individual materials and presenting them with the various positions of the actors in their texts [24] (p. 29).

Metacognitive strategy use would have been particularly helpful for mastering the task’s requirements (i.e., understanding that collecting information from the material is necessary in order to write a convincing argumentative text, see Appendix A; the respective metacognitive strategy is the fifth subtype of *M/C UIT*). Considering this, we interpret that a relationship exists within our sample, in that our students did not use any metacognitive strategies during their work process to manage how they filtered and understood the information and, consequently, they only referred to very few of the materials in their texts. The opposite also seems to exist, as a positive correlation seems to exist between a strategic and goal-oriented working process that includes metacognitive strategies in material-based argumentative writing and a better-quality final text product. An example of this was the results from S20, who produced the strongest text (77 Pts.) and used the most metacognitive strategies (24 in total). Here, particularly in the areas of *M/C UIT* (*Monitoring/Controlling Understanding Initiating Task*), *M/C GS* (*Monitoring/Controlling Goal(s) Setting*), and *M/C PL* (*Monitoring/Controlling Planning*), S20’s use of metacognitive strategies was particularly useful in the formulation of their text.

Looking at what and how many occurrences of metacognitive activity can be reconstructed from the think-aloud protocols for each of the 18 students in more detail, we observed that in the area of *M/C GS*, the three learners (S16, S17, S20) who thought about their writing goals and relevant actions wrote higher quality texts compared to their classmates. S16’s text scored 49 points, S17’s text achieved 48 points, and S20 submitted the strongest text of the group, with 77 points (see Figure 3). However, these three students did not think about the primary goals concerning the content of the text to be written (i.e., naming conflict or actors), instead focusing on the type of text (statement) (i.e., S20: “I will now write down my statement”) or the order of their actions (i.e., S16: “Now I’ll take a look at the next tasks” or S17: “This is a long text. Maybe I will read it in a moment”). Similar results were found in the area of *M/C PL*. The texts of most of the students who did not use any or only very few planning strategies (especially, i.e., S1SLD/ED, S2, S6, S8, S14, and S18SLD) were of poor quality, whereas for example, S20, who undertook the most metacognitive planning activities by far, presented the strongest text. S3, S6, and S14 performed no strategy use at all during the working process and their texts received low scores (see Figure 3).

Overall, in our sub-study on metacognition, we were able to determine a positive correlation coefficient (Pearson correlation coefficient = 0.50) for the relationship between metacognitive strategy use and text quality that was statistically significant (*p* = 0.03). The strongest positive correlation coefficients were identified for *M/C GS* (*Monitoring/Controlling Goal(s) Setting)* (0.79) and *M/C PL* (*Monitoring/Controlling Planning*) (0.67).

## 4. Discussion

Bringing together all of the results of the sub-study, the following main points regarding the use of metacognitive strategies in material-based argumentative writing can be raised for discussion:

1. Predominant tendency: hardly any planning of the work processes:

None of the students undertook a full strategic and supervised planning and execution approach to the working process. A small number of students planned single aspects of the process, but these planning processes were not profound, and the written texts were not revised after writing. When it comes to planning strategies (*M/C PL*), the students’ considerations lacked sufficient depth when naming their next working steps, not describing them further with regard to their execution. Altogether, strategies involving the systematic setting of task-oriented goals (*M/C GS*) as well as considerations addressing the revision of the final text (*M/C REV*) were used the least. If we assume, as Ahmadi et al. [10] do, that planning activities already constitute an essential area already of metacognitive *reading* strategies, and if we also follow Baker [13] as well as Kraayenoord [17] in recognizing that metacognition (in reading processes) only develops during adolescence, then our data suggest that the essential area of planning activities is still underdeveloped at younger ages. The predominant non-existence of planning processes may also be a consequence of the fact that the task itself was not understood in a deeper way (see the next point)

2. Predominant tendency: incomplete understanding of the task:

The students either did not reflect on the task requirements at all or not to the full extent (*M/C UIT*). This result suggests that their understanding of the task was incomplete and unsystematic from the beginning of the working process. This result is also supported by their argumentative texts being deficient in the main aspects and requirements of the task. Again, if we agree with Kraayenoord that “[…] good comprehenders are more aware than poor comprehenders” [17] (p. 284), then our results are in line with these research findings.

3. Predominant tendency: focus on metacognitive activities expressing comprehension problems and on (superficial) design elements:

From the think-aloud protocols, half of the students (nine in total) demonstrated metacognitive activities when reading the reference material (*M/C RD*). However, most of their reflection considerations focused on expressing comprehension problems, which were accepted by the students without considering solutions to solve the discovered difficulty. This again fits with Goldman et al.’s [21] observations as well as with Anmarkrud et al.’s [22] that it is relevant to not only notice comprehension problems, but to monitor them more deeply and to regulate one’s approach to the task. Moreover, the students did not evaluate the material with regard to the extraction of useful information and instead tended to evaluate the materials in terms of its design. This is comparable to the study of Meneghetti et al. [18], which highlighted that the key, complex metacognitive skills involve, among other things, the differentiation of relevant and non-relevant information as well as the adaptation of reading strategies for the respective text type.

4. If formulations were reflected in a few cases, then with reference to the material:

The majority of the students did not reflect on the formulation process of the text to be written (*M/C FL*). However, those who executed thoughts about their formulations focused on the requirement to embed material-based information into the text and on the search for and choice of appropriate formulations to do so.

Possible reasons for the use of very few metacognitive strategies by the students are (i), following Baker [13] as well as Kraayenoord [17], the early time of engagement with the task (8th grade of a comprehensive school/German Gesamtschule), (ii) an unfamiliar teaching setting, but, most of all, (iii) a lack of these strategies, which is probably due to a lack of engagement with metacognitive strategies in class. When analyzing the results of the study, it must be also taken into account that linguistic comprehension and the mastery of vocabulary in particular was a challenge for many students. Additionally, verbalizing thoughts in the context of think-aloud protocols was an unfamiliar activity for the subjects and was associated with feelings of shame for some subjects.

5. Positive correlation between the use of metacognitive strategies and a better text product:

Finally, there was a positive correlation between the use of metacognitive strategies and a better text product. A lack of metacognitive strategies may thus explain students’ poor performance in producing material-based argumentations. Overall, the positive effects of metacognition on students’ classroom performance that have been suggested in the literature [6,22,23] are confirmed in our sub-study.

It is important to note that these results and interpretations were drawn from a small sample and that the results therefore may not be representative. However, they do allow for well-founded hypotheses. In this respect, we claim that it is not only the quantity of used strategies that is important, but also the quality with which each strategy is applied. Thus, solution-oriented strategies such as planning work processes prove to be more useful than simply naming difficulties.

## 5. Conclusions

Unlike most previous studies, the presented study did not focus on metacognitive processes in either reading or writing, but looked at both domains in relation to each other. Since working with multiple sources and especially with argumentative writing based on multiple materials is a school task format that addresses the demands of modern societies, especially in the era of digital transformation, a deeper understanding of how reading and writing processes occur in the performance of this task and in the role of metacognition is necessary. Further insights are also needed in terms of empowering students to perform this challenging task.

With regard to the task format of writing using multiple sources, it was observed that the use of metacognitive strategies when reading and writing had a positive effect on the quality of the produced texts in our group of test students. If one considers the use of metacognitive strategies as a relevant prerequisite for writing successful arguments, then the systematic training of these strategic skills, which can help students to better control their work processes and thus be more focused as well as more successful is recommended. For writing in general, the self-regulated strategy development (SRSD) model developed by Harris and Graham (1996) [1] is now well established, and its effectiveness well studied, especially in weaker writers [33,34,35]. However, there are few didactic proposals to date that specifically address the promotion of metacognitive strategies in writing using multiple sources. As we were able to show with this research, the support must refer to all areas mentioned in the model (Figure 1). There is therefore a need for the development and evaluation of suitable didactic concepts going forward.

## Figures and Tables

**Table 1:** Extract of the coding guide for the empirical reconstruction of metacognitive strategies in argumentative writing based on multiple sources (own elaboration).

Research Question: Which Metacognitive Strategies Do Students Use in Argumentative Writing Based on Multiple Sources?						
No.	Process Step of Argumentative Writing Based on Multiple Sources	Main Category of Metacognitive Strategy		No.	Related Subtype of Metacognitive Strategy	Exemplary Occurrences ^1^ in the Think-Aloud Protocols
**1**	**Understanding Initiating Task**	**Monitoring/Controlling** **understanding of the initiating task** Explanation: The student evaluates one’s own understanding of the overall task as a complex task by identifying and confirming the subtasks.	**M/C UIT**	1	The student thinks about what they need to do to complete the overall task.	S2: (Explaining the task to a classmate): *Exactly*, *this question we have to answer.*
				2	The student recognizes they are supposed to develop their own opinion and to integrate it in the text.	S19: *Now I’m supposed to write what I´m thinking about it, I´m supposed to write whether I’m for it or against it.*
				3	The student recognizes that they are supposed to justify their own opinion based on arguments.	S1 SLD/ED: *I´ve justified my opinion.*
				4	The student recognizes that they are supposed to read/evaluate the material for the development of an own opinion and its integration in the text.	S16: (Speaking to a classmate): *You also have to read this diagram here.*
				5	The student recognizes that they are supposed to find information and arguments from the material and use them in the text.	S11: *So, I´m collecting counterarguments and arguments in favor of it.*
**2**	**Goal(s) Setting**	**Monitoring/Controlling** **of the goal setting for overall task processing** Explanation: The student sets goals for the processing of the overall task, including for the subtasks and structures.	**M/C GS**	4	The student reflects on the sequence of one’s own actions and defines an order for the working steps.	S20: *Now I’ve gone through all the sheets and now I’ll look at the task again.*
**3**	**Reading**	**Monitoring/Controlling** **the reading/reception of the sources/material** Explanation: The student reflects on the steps involved in reading and evaluating each source.	**M/C RD**	1	The student thinks about reading strategies (and decides which reading strategy to use for a particular material).	S7: *I´ll look through all the pages again.* (Student is reading the material again).
				3	The student evaluates the material in terms of its relevance for the fulfillment of the task.	S16: *I don’t really think anything of the diagram, because it’s actually about the issue with the training grounds and that has very little to do with it.*
**4**	**Planning**	**Monitoring/Controlling** **the planning of writing** Explanation: The student reflects on the planning/structuring of the writing process and on the composition and structure of the text product.	**M/C PL**	1	The student thinks about the order and related steps for writing the text.	S13: *I´ll now write down what I´ve thought throughout the conversation and will also read through the sheets now and then.*
				4	The student recognizes and identifies problems in the planning process.	S12: *I don’t want to write a whole novel now, as I usually do in Geography lessons, because then I digress from the topic and that would no longer be appropriate for the task requirement.*
**5**	**Formulating**	**Monitoring/Controlling** **the formulating** Explanation: The student reflects on the written communicative actions and the formation of the text product.	**M/C F**	1	The student reflects on the appropriate use of written argumentative procedures.	S11: *How should I phrase this?*
				3	The student reflects on the grammatical and orthographical correctness of the writing (and considers the use of appropriate strategies to correct discovered errors and uncertainties (e.g., dictionary, smartphone, asking the instructor)).	S12: *Oh God, my spelling.*
				4	The student recognizes and identifies problems in the formulation process.	S12: *Oh, oh, writing blockade. What else should I write? Full stop. What should I write? I’m kind of pressed for time right now, and I didn’t want to be pressed for time.*
**6**	**Reviewing**	**Monitoring/Controlling** **the reviewing** Explanation: The student reflects on steps in text revision.	**M/C REV**	2	The student checks content and formulations of the resulting text and possibly revises them.	S9 ED: *I´m finished. I couldn´t erase it, is that bad?* (Instructor is replying that it is no problem). *Can you read it?* (Instructor is confirming that they can read it).

^1^ The original verbalizations from the German students were translated into English by the authors.

## Data Availability

The data that support the findings of this study are available from the corresponding authors, upon reasonable request.

## Appendix A. Coding Guide for Empirical Reconstruction of Metacognitive Strategies in Argumentative Writing Based on Multiple Sources (*Own Elaboration*)

**Table:**

Research Question: Which Metacognitive Strategies Do Students Use in Argumentative Writing Based on Multiple Sources?						
No.	Process Step of Argumentative Writing Based on Multiple Sources	Main Category of Metacognitive Strategy		No.	Related Subtype of Metacognitive Strategy	Exemplary Occurrences in the Think-Aloud Protocols
**1**	**Understanding Initiating Task**	**Monitoring/Controlling** **understanding of the initiating task** Explanation: The student evaluates her/his understanding of the overall task as a complex task by identifying and concretizing the subtasks.	**M/C UIT**	1	The student thinks about what is necessary to do to complete the overall task.	S2: (Explaining the task to a classmate): *Exactly*, *this question we have to answer.*
				2	The student recognizes that they are supposed to develop their own opinion and to integrate it in the text.	S19: *Now I’m supposed to write what I’m thinking about it, I’m supposed to write whether I’m for it or against it.*
				3	The student recognizes that they are supposed to justify their own opinion based on arguments.	S1 SLD/ED: *I’ve justified my opinion.*
				4	The student recognizes that they are supposed to read/evaluate the material for the development of their own opinion and its integration into the text.	S16: (Speaking to a classmate): *You also have to read this diagram here.*
				5	The student recognizes that they are supposed to find information and arguments in the material and use them in the text.	S11: *So, I’m collecting counterarguments and arguments in favour of it.*
**2**	**Goal(s) Setting**	**Monitoring/Controlling** **of the goal setting for overall task processing** Explanation: The student sets goals for the processing of the overall task with its involved subtasks and structures.	**M/C GS**	1	The student determines the goal of identifying the conflict when working through the material and presenting the conflict in the text.	-
				2	The student determines the goal of identifying the different actors and positions of the conflict when working through the material and presenting them in the text.	-
				3	The student determines the goal of writing their text in an addressee- and goal-oriented manner and thus realizes the intended text function (letter/statement)	S20: *I will now write down my statement*
				4	The student reflects on the sequence of one own’s actions and defines an order for the working steps.	S20: *Now I’ve gone through all the sheets and now I’ll look at the task again.*
**3**	**Reading**	**Monitoring/Controlling** **the reading/reception of the sources/material** Explanation: The student reflects on the steps involved in reading/evaluating each material.	**M/C RD**	1	The student thinks about reading strategies (and decides which reading strategy to use for a particular material). (i.e., adapting one’s reading behavior to the type of text, selective reading, looking for more information, note taking, underlining, determining an order when reading texts, reading headings first, identifying graphic elements, reading the caption first for maps/statistics)	S7: *I’ll look through all the pages again.* (Student is reading the material again).
				2	The student selects evaluation strategies. (i.e., reflecting on locating/extracting/combining (cf. PISA 2018) information/arguments relevant for the task)	S12: *Should I write anything down about this? So, I can’t write anything down yet? (Instructor: Asking whether S12 means note-taking?) No, I can’t write anything down here.*
				3	The student evaluates the material in terms of its relevance for the fulfillment of the task.	S16: *I don’t really think anything of the diagram, because it’s actually about the issue with the training grounds and that has very little to do with it.*
				4	The student reflects on the credibility of the source, the subjectivity/personal interests (cf. PISA 2018).	S2: *Here I see a picture, M8. This looks designed with Photoshop.*
				5	The student recognizes (and solves) problems in the reading process.	S13: *I’m reading right now, there are words I don’t understand, like NABU.*
				6	The student reflects on linguistic expressions. (i.e., direct/indirect meaning, irony, other rhetorical features, etc.)	S8: *One is against it one is for it. And I think that they’re having an argument, as you can see here, because there are a lot of speech bubbles or thought bubbles (…).” In addition, S8 also refers to typographical highlighting of lexical entities in the speech bubbles by explaining: “(…) there are a lot of speech bubbles or thought bubbles, even capitalized once. So a little attentive, I think, and underlined.”*
				7	The student reflects on the need to compare relevant information, to check it for contradictions and repetitions, and to consider the hierarchy of information/arguments.	-
**4**	**Planning**	**Monitoring/Controlling** **the planning of writing** Explanation: The student reflects on the planning/structuring of the writing process and on the composition and structure of the text product.	**M/C PL**	1	The student thinks about the order and related steps for writing the text. (i.e., first collect all arguments; then start writing; during writing, include phases of going back to the material, searching for information, and checking sources, writing everything down, and then reading through the whole text again or stopping writing in between and reading through parts of the text, etc.)	S13: *I’ll now write down what I’ve thought throughout the conversation and will also read through the sheets now and then.*
				2	The student plans the text on a structural level. (i.e., global text structure (introduction, main part, conclusion), the argumentative structure on a general level (own opinion first and then argumentation or vice versa?), the formal structure of the argumentation (sand glass or ping-pong method?)	S11: *How should I start? What I’m thinking when I’m writing? Simply my own opinion.*
				3	The student plans the text on the content level. (i.e., argumentation on the content level (selection, sequence, and linking of arguments), establishing references to the material (use of quotations, references to sources), considering the intention and addressee of the text)	S11: *So, I’m collecting counterarguments and arguments in favor of it.*
				4	The student recognizes and identifies problems in the planning process.	S12: *I don’t want to write a whole novel now, as I usually do in Geography lessons, because then I digress from the topic and that would no longer be appropriate for the task requirement.*
**5**	**Formulating**	**Monitoring/Controlling** **the formulating** Explanation: The student reflects on the written communicative actions and the formulations of the text product.	**M/C F**	1	The student reflects on the appropriate use of written argumentative procedures. (i.e., reflecting on own their formulations, thinking about adopting formulations from the reference material, reflecting on decisions for/against formulations, looking for help (dictionary, smartphone, asking the instructor))	S11: *How should I phrase this?*
				2	The student reflects on the appropriateness of his language (registering) in alignment with the text type (letter/statement).	-
				3	The student reflects on the grammatical and orthographical correctness of the writing (and considers the use of appropriate strategies to correct discovered errors and uncertainties (e.g., dictionary, smartphone, asking the instructor)).	S12: *Oh God, my spelling.*
				4	The student recognizes and identifies problems in the formulation process.	S12: *Oh, oh, writing blockade. What else should I write? Full stop. What should I write? I’m kind of pressed for time right now, and I didn’t want to be pressed for time.*
**6**	**Reviewing**	**Monitoring/Controlling** **the reviewing** Explanation: The student reflects on the steps required for text revision.	**M/C REV**	1	The student reflects on the essential steps of the review process. (i.e., what to do when revising the text, reading through the text product again, how to revise the text if something is missing in terms of content or morpho-syntax, and what to do if parts of the text are not good)	-
				2	The student checks content and formulations of his text and possibly revises them.	S9 ED: *I’m finished. I couldn’t erase it, is that bad?* (Instructor is replying that it is no problem). *Can you read it?* (Instructor is confirming that you they read it).
				3	The student checks whether the intended text effect/function has been achieved.	
